# Ocular and Systemic Risk Factors of Different Morphologies of Scotoma in Patients with Normal-Tension Glaucoma

**DOI:** 10.1155/2017/1480746

**Published:** 2017-07-26

**Authors:** Ewa Kosior-Jarecka, Dominika Wróbel-Dudzińska, Urszula Łukasik, Tomasz Żarnowski

**Affiliations:** Department of Diagnostics and Microsurgery of Glaucoma, Medical University of Lublin, Lublin, Poland

## Abstract

**The Aim:**

The aim of this study was to assess general and ocular profiles of patients with single-localisation changes in visual field.

**Material and Methods:**

The study group consisted of 215 Caucasian patients with normal-tension glaucoma with scotoma on single localisation or with preperimetric glaucoma. During regular follow-up visits, ophthalmic examination was carried out and medical history was recorded. The results of the visual field were allocated as paracentral scotomas, arcuate scotomas, peripheral defects, or hemispheric defects. Statistical analysis was conducted with Statistica 12, and *p* < 0.05 was considered statistically significant.

**Results:**

Risk factors such as notch, disc hemorrhage, general hypertension, migraine, and diabetes were strongly associated with specific visual field defects. Paracentral defect was significantly more frequent for women (*p* = 0.05) and patients with disc hemorrhage (*p* < 0.001). Arcuate scotoma occurred frequently in patients without disc hemorrhage (*p* = 0.046) or migraines (*p* = 0.048) but was observed in coexistence with general hypertension (*p* < 0.001). The hemispheric defect corresponded with notch (*p* = 0.0036) and migraine (*p* = 0.081). Initial IOP was highest in patients with arcuate scotoma and lowest in patients with preperimetric glaucoma (*p* = 0.0120).

**Conclusions:**

The specific morphology of scotoma in patients with normal-tension glaucoma is connected with definite general and ocular risk factors.

## 1. Introduction

Normal-tension glaucoma is a subtype of primary open-angle glaucoma where maximal intraocular pressure (IOP) never exceeds 21 mmHg [1]. Some studies did not differentiate normal-tension glaucoma (NTG) from high-tension glaucoma (HTG) finding it a continuum of the same disease. IOP remains well recognized and the only modifiable factor in pathogenesis and treatment of primary open-angle glaucoma [2]. In glaucoma with initial IOP within the normal range in the absence of this pathogenic factor, the other causative agents need consideration. The risk factors for development and progression of normal-tension glaucoma are associated with general status as low blood pressure [3], migraines [4], dysregulation of blood flow [5], and diabetes mellitus [6]. Additionally, there are studies showing that NTG has some ocular indicators such as disc hemorrhages [7], parapapillary atrophy [8], and low central corneal thickness [9].

An interesting issue are visual field scotomas described in NTG patients. It has been reported that visual field defects are more likely to be deeper, steeper, and closer to fixation in NTG compared to HTG [10, 11]. The characteristics of visual field changes in NTG have been reported as the occurrence of dense scotomas close to fixation and often at low levels of total loss, whereas in primary open-angle glaucoma (POAG) with typical arcuate scotoma at Bjerumm's region, paracentral scotomas tend to occur in association with a more advanced field loss [12].

The aim of this study was to evaluate general and ocular profile of patients with specific visual field (VF) changes in the absence of elevated IOP.

## 2. Material and Methods

The studied group included in this retrospective analysis consisted of 215 Caucasian patients with NTG (151 females and 64 males) treated at the Department of Diagnostics and Microsurgery of Glaucoma of the Medical University of Lublin, Poland, between 2012 and 2015. Clinical characteristics of the group were shown in Table 1. Only the patients with scotoma on single localisation or with preperimetric glaucoma were recruited. Preperimetric glaucoma was diagnosed according to disc morphology and RNFL OCT measurements (Cirrus, Zeiss).

Written consent was obtained from all patients before their enrolment in the study and was included in each patient's study documentation. The study adhered to the tenets of the Declaration of Helsinki, and the study design was approved by the Ethics Committee of the Medical University of Lublin (approval number: 127/12).

Patients with NTG met the following inclusion criteria: a glaucomatous neuroretinal rim loss, an open angle under gonioscopy, and IOP consistently <21 mmHg (the highest measured before introduction of antiglaucoma therapy was ≤21 mmHg and was called maximum IOP). During regular follow-up visits, best-corrected visual acuity (BCVA), maximum IOP, and IOP with Goldmann applanation tonometry were measured, and gonioscopic, pachymetric, visual field (VF) (30-2 SITA-fast, Humphrey), biomicroscopy, and stereoscopic fundus examinations were carried out. Of all the VF data, we excluded the unreliable VF results (fixation loss > 20%, false positive and false negative > 15%). All IOP measurements were corrected according to measurements of central corneal thickness (CCT) obtained during pachymetry. Medical history was recorded regarding glaucoma, other ophthalmic diseases, chronic general disorders, and vascular risk factors (migraine, low blood pressure, and cold extremities).

The results of the visual field examinations were allocated as paracentral scotomas, arcuate scotomas, peripheral (nasal/temporal step) defects, or hemispheric defects as described previously [13, 14]. Patients with different morphologies of the scotoma in the right and left eye were excluded from the study. While testing the ocular factors, each eye was counted separately; while considering general risk factors, they were assessed for each patient.

Statistical analysis of the results was conducted with the Statistica 12 software, and *p* < 0.05 was considered statistically significant. The results were reported mainly as mean ± SD or percentage values. Normal distribution was checked with Shapiro-Wilk test. We used *t*-test to compare normally distributed data and in case of non-normally distributed data ANOVA Kruskal-Wallis test with Tukey as post hoc test. Chi-square test with Yates modification when needed was used to check the association between risk factors of glaucoma and visual field defects. In case of statistical significance obtained in chi-square test, logistic regression analysis was performed to assess odds ratios (OR). Correlations were assessed using Spearman test.

## 3. Results

Two hundred eighty eyes of 215 subjects were included in the study. Demographic and clinical characteristics of the whole studied group was shown in Table 1. Preponderance of women was observed in the studied group.

After dividing patients according to the localisation of scotoma, no differences were found in age (*p* = 0.8561), age of diagnosis (*p* = 0.6255), BCVA (*p* = 0.1880), and CCT (*p* = 0.7201) in patients with different visual field features (Table 2).

The highest initial IOP was observed in patients with arcuate scotoma and the lowest in patients with preperimetric glaucoma (*p* = 0.0120; arcuate versus preperimetric: *p* = 0.0276). There was no correlation between intraocular pressure and MD at diagnosis (*p* = 0.529). We did not observe any correlation between initial intraocular pressure and age of diagnosis (*p* = 0.18).

In chi-square analysis, paracentral defect was significantly more frequent in women (*p* = 0.05) and patients with disc hemorrhage (*p* < 0.001) but less frequent in patients with diabetes mellitus (*p* = 0.0168). Early arcuate scotoma occurred less frequently in patients with disc hemorrhage (*p* = 0.046) and migraines (*p* = 0.048), but it was more frequent in patients with general hypertension (*p* < 0.001). Hemispheric defect was observed in patients with notches (*p* = 0.0036) and migraine (*p* = 0.081) but rarer in patients with disc hemorrhages (*p* = 0.0815). In patients with a preperimetric form of normal-tension glaucoma, female gender (*p* = 0.0221) and general hypertension (*p* < 0,001) were observed less frequently. The details of the data are put in Tables 2 and 3.

The results of logistic regression analysis were shown in Table 4. Paracentral scotoma was 3.98 more frequent in patients with disc hemorrhages. Disc hemorrhages were less frequent in hemifield defect (OR = 0.28) and in preperimetric NTG (OR = 0.44).

## 4. Discussion

Primary open-angle glaucoma (POAG) involves optic nerve fiber layer dropout that produces a visual field (VF) loss, which has heterogeneous patterns. The optic nerve contains distinct retinal ganglion cell populations with different vulnerabilities [14, 15], so the etiology of POAG may differ in diverse patterns of early VF loss. Early scotomas in glaucoma are typically found in Bjerumm's region 10–20 degrees from the fixation point, thus sparing central visual acuity until the advanced stage of the disease, but some eyes develop defects threatening fixation in the earlier stages of glaucoma with a consequent greater risk of the reduced quality of life. Patients with a glaucomatous paracentral VF loss may be at greater risk of losing useful visual acuity [16]. Additionally, a paracentral VF loss causes problems in the performance of daily life activities by difficulty in reading [17] and worsened driving performance [18]. In this study, typical arcuate scotoma was the most frequent type of early changes in VF in normal-tension glaucoma. However, paracentral defect was present in 25% of patients with NTG, which was twice more frequent comparing to that of Polish patients with primary open-angle glaucoma with high initial IOP [19].

According to the results of our study, every pattern of observed single-site changes in visual field has specific features. Risk factors for paracentral defects are female gender and disc hemorrhages, but diabetes seems to be protective. The results of our study did not confirm the influence of migraines, cold extremities, or general hypotension as suggested in some studies [20]. Patients with arcuate scotoma had more frequently general hypertension, with low occurrence of migraines and disc hemorrhages. In case of hemispheric defects the lowest initial IOP, notches on the disc and migraines were observed, but disc hemorrhages were rare. Patients with no changes in VF were more frequently males with lower IOP and without DH or general hypertension.

Normal-tension glaucoma is believed to be at least partially IOP dependent. There are studies which show the influence of intraocular pressure on different aspects of NTG. Lee et al. [21] demonstrated that VF deterioration increases 4.2-fold for each 1 mmHg increase in IOP in patients with POAG including NTG and ocular hypertension. According to the results of EMGT, the mean rate of VF deteriorations −1.31 dB/year in the higher initial IOP group compared to −0.36 in dB/year in the lower baseline IOP group [22]. This is in concordance with the results of this study where IOP differentiates patients with no scotoma who tend to have lower IOP from the group with changes in VF (in case of arcuate scotoma, this difference is statistically significant) with the exception of hemispheric defect, which developed in patients with low baseline IOP. Additionally, it is claimed that NTG eyes progress more often in the center [23] and that central worsening of scotoma may have significant association with IOP during the follow-up period [24]. In our study, initial IOP of patients with paracentral defect was similar to the one observed for arcuate and peripheral scotomas (as in Rao and Mukherjee [25]), which stays on the contrary to the results of some studies [15, 26].

In our patients with early NTG, there was a clear preponderance of women which was observed by the others [27]. Female gender was also more frequent in the case of paracentral visual loss. It may indicate that there are factors associated with female gender influencing the course of the disease. Some hypotheses concerning female preponderance suggest an association between the estrogen SNP pathways and POAG among females [28]. But there may be other biological mechanisms underlying the observed gender specificity such as epigenetic differences and possible neuroprotective action of sex hormones [29].

Disc hemorrhage (DH) is a feature of glaucomatous optic neuropathy commonly observed in NTG and rarely found in normal eyes [30]. The pathogenesis of DH remains unclear. It develops on the level of the lamina cribrosa where there are four biomechanical forces: IOP, arterial pressure, venous pressure, and cerebrospinal fluid pressure. The occurrence of DH may be associated with pathologic changes or an imbalance of these forces [31]. In this study, DH were more than 3.5 times frequent in patients with paracentral scotoma being not only the predictor of progression as suggested by some authors [32–34] but also the indicator of localisation of the defect in the paracentral region of VF and endangerment of an early visual loss, which was described previously by Kang et al. for the Korean population [35].

Primary vascular dysregulation with general hypotension, migraines, and cold extremities, which destabilises perfusion pressure in the eye, was described by some authors with higher frequency in paracentral scotomas [24, 35]. We could not confirm this for the Polish population, which may be connected with different ethnic specificity as described for Raynaud's phenomenon [36]. The frequency of migraines observed in our group was similar to those of a general population [37]. Additionally, diabetes mellitus, one of the main causes of secondary vascular dysregulation, was less frequent in patients with paracentral scotomas. Similar to our results, in the recent study of visual field profiles for POAG, early peripheral scotoma, as opposed to paracentral scotoma, a visual field loss, was more common in POAG patients with diabetes mellitus [14].

Barbados Eye Study [38] reported that hypertension has a protective role in development of glaucoma by maintaining an adequate perfusion for the optic nerve. However, according to the results of the Blue Mountains Eye Study, hypertension was significantly associated with OAG and this relation was strongest in subjects with poorly controlled treated hypertension [39]. In the Rotterdam Study, systemic blood pressure and hypertension were associated with IOP and high-tension glaucoma but no association was found between blood pressure or hypertension and NTG [40]. In our study, general hypertension was twice more frequent in patients with arcuate scotoma. Interestingly, this group had also the highest values of initial IOP. It may indicate that arcuate scotoma, typical for glaucoma, is mainly IOP dependent. In our study, general hypertension was not a risk factor in paracentral or peripheral scotoma as described previously [14, 15, 35, 41].

Ischemia due to periodic vasoconstriction as described during migraines is considered as a potential risk factor for glaucomatous visual field damage [37]. In our group, migraines were more frequent in patients with hemispheric defect. Some authors speculate that initial paracentral scotomas during progression become a hemifield defect [42], but all patients with a hemifield defect in our study had this morphology of scotoma since the time of diagnosis. In the last study of NTG and POAG eyes with hemifield structural and functional defects, vessel diameter was not significantly different in the affected and unaffected quadrants, which may point toward the possibility that vascular diameter changes are not the cause for glaucomatous changes [43]. In the study characterizing the visual fields of subjects with migraine headaches, many of the visual field deficits observed were present in the peripheral visual field and were similar to the arcuate type of deficits reported for early stages of glaucoma [44].

According to the results of this study, it is possible to hypothetically predict the morphology of early scotoma appearing in the visual field by taking into consideration the general and ocular profile of a patient with normal-tension glaucoma. It could potentially be helpful in identifying the patients endangered with an early loss of useful visual acuity because of paracentral scotoma.

## Figures and Tables

**Table 1:** Demographic characteristic of the studied group.

	Studied group
Number of patients	215
Number of eyes	280
Females	151 (70%)
	212 eyes (76%)
Mean age	70.5 ± 10
Time from diagnosis	4.6 ± 4.9 years
BCVA (mean)	0.7
Maximal IOP (mmHg)	17.3 ± 2.6
CCT (*μ*m)	537 ± 36.9
CDR (mean)	0.76 ± 0.12
PPA (number of eyes)	69
Notch (number of eyes)	129
Disc hemorrhages (number of eyes)	101
Diabetes mellitus	70
Migraines	44
Hypertonia arterialis	104
Cold extremities	60
General hypotension	46

**Table 2:** Ocular characteristics of the subgroups of visual field defects. In the table, a number of eyes were shown; a chi-square test compared if the specific feature is more/less frequent in the group with specific morphology of scotoma comparing to that in the rest of the studied group.

	Paracentral	Arcuate	Peripheral	Hemispheric	Preperimetric
Number of eyes	70	93	28	23	66
Number of eyes of women	59	70	21	19	43
	*p* = 0.0535	*p* = 0.9024	*p* = 0.8892	*p* = 0.8454	*p* = 0.0221^∗^
Age (years)	71	71.3	66.8	66.1	71.6
Maximal IOP (mmHg)	17.7	17.8	17.8	16.5	16.3
CCT (*μ*m)	534	534.8	544.6	542.3	540.5
Parapapillary atrophy	13	27	7	5	17
	*p* = 0.0989	*p* = 0.4261	*p* = 0.8917	*p* = 0.9051	*p* = 0.9470
Notch	29	44	11	17	28
	*p* = 0.2402	*p* = 0.9645	*p* = 0.4594	*p* = 0.0036^∗^	*p* = 0.3449
Disc hemorrhage	41	26	16	4	14
	*p* = 0.0000^∗^	*p* = 0.046^∗^	*p* = 0.14	*p* = 0.0161^∗^	*p* = 0.0004^∗^

^∗^Statistically significant.

**Table 3:** General risk factors in studied subgroups. In the table, a number of eyes were shown; a chi-square test compared if the specific risk factor is more/less frequent in the group with specific morphology of scotoma comparing to that in the rest of the studied group.

	Paracentral	Arcuate	Peripheral	Hemispheric	Preperimetric
General hypertension	26	52	7	10	9
	*p* = 1.0	*p* = 0.0000^∗^	*p* = 0.4871	*p* = 0.5116	*p* = 0.0000^∗^
General hypotension	15	12	4	4	11
	*p* = 0.1924	*p* = 0.2616	*p* = 0.9571	*p* = 0.87	*p* = 0.9524
Migraines	9	10	4	7	14
	*p* = 0.2171	*p* = 0.0438^∗^	*p* = 0.8528	*p* = 0.0815	*p* = 0.94
Diabetes mellitus	10	24	8	8	20
	*p* = 0.0168^∗^	*p* = 0.826	*p* = 0.6037	*p* = 0.2831	*p* = 0.1886
Cold extremities	10	20	9	3	18
	*p* = 0.1301	*p* = 0.9827	*p* = 0.2249	*p* = 0.4486	*p* = 0.1857

^∗^Statistically significant.

**Table 4:** Logistic regression analysis of morphology of the scotoma with risk factors.

Morphology of scotoma	Risk factor	*p* value	Odds ratio (OR)	Confidence interval (CI)
Paracentral	DH	0.0000^∗^	3.98	−95%CI = 2.25
				+95%CI = 7.03
	DM	0.18	0.5	−95%CI = 0.18
				+95%CI = 1.39

Arcuate	DH	0.103	0.64	−95%CI = −0.38
				+95%CI = 1.09
	General hypertension	0.058	2.01	−95%CI = −0.97
				+95%CI = 4.16
	IOP	0.056	1.11	−95%CI = 0.99
				+95%CI = 1.24
	Migraine	0.54	0.78	−95%CI = 0.35
				+95%CI = 1.74

Hemifield	DH	0.046^∗^	0.28	−95%CI = 0.08
				+95%CI = 0.97
	Notch	0.016^∗^	4.75	−95%CI = 1.33
				+95%CI = 16.75
	Migraine	0.09	2.47	−95%CI = 0.85
				+95%CI = 7.12

Preperimetric	DH	0.013^∗^	0.44	−95%CI = 0.23
				+95%CI = 0.84
	General hypertension	0.09	1.89	−95%CI = 0.89
				+95%CI = 4.03
	IOP	0.0036^∗^	0.83	−95%CI = 0.74
				+95%CI = 0.94

DH: disc hemorrhages; DM: diabetes mellitus; IOP: intraocular pressure. ^∗^Statistically significant.
